# Trends in ischemic heart disease-related mortality in obese population in the United States

**DOI:** 10.1097/XCE.0000000000000325

**Published:** 2025-03-03

**Authors:** Shehroze Tabassum, Faraz Azhar, Fatima Hussain, Aroma Naeem, Mohammad Ali Sheffeh, Muhammad Sohaib Asghar

**Affiliations:** a Department of Internal Medicine, King Edward Medical University; b Department of Internal Medicine, Allama Iqbal Medical College, Lahore; c Department of Internal Medicine, Dow International Medical College, Karachi, Pakistan; d Department of Internal Medicine, The Wright Center for Graduate Medical Education, Scranton, Pennsylvania; e Department of Internal Medicine, Henry Ford Warren Hospital, Warren, Michigan; f Department of Cardiovascular Medicine, Mayo Clinic Rochester, Rochester, Minnesota; g Department of Internal Medicine, AdventHealth Sebring, Sebring, Florida; h Division of Nephrology and Hypertension, Mayo Clinic Rochester, Rochester, Minnesota, USA

**Keywords:** Ischemic heart disease, Obesity, Mortality, Trends, Epidemiology

## Abstract

Obesity affects approximately 72 million Americans and is a significant contributor to ischemic heart disease (IHD). Given the scarcity of data, this observational study examines trends and disparities in IHD-related mortality among obese individuals in the United States from 2003 to 2019 using Centers for Disease Control and Prevention’s Wide-Ranging Online Data for Epidemiologic Research data. Age-adjusted mortality rates (AAMRs) were calculated for IHD as the underlying cause of death and obesity as a contributing cause of death, revealing an increase in IHD-related mortality among obese adults. AAMR rose from 2.1 in 2003 to 3.9 in 2019, with higher rates in men, non-Hispanic Black individuals, the elderly, and those in nonmetropolitan and Midwest regions. These findings underscore significant sex, racial, and regional disparities in mortality, suggesting a need for targeted health policies and resource allocation, improving overall cardiovascular health outcomes.

Obesity remains a major health issue in the United States (US), currently affecting approximately 72 million people [1]. Forty years ago, almost 25% of US adults were classified as overweight or obese; however, this number has escalated to 70% at present, making obesity a new epidemic [2]. Obesity is a well-recognized and substantial risk factor for ischemic heart disease (IHD), and the simultaneous presence of these conditions intensifies adverse health outcomes and hinders treatment approaches. While the association between IHD and obesity is well-documented, there is a significant disparity in mortality rates from IHD among obese individuals in the US across several geographical and demographic locations [3]. Investigating the patterns and inequalities in IHD-related death rates among obese individuals is vital for designing targeted approaches to alleviate the impact of these interconnected health conditions. This retrospective observational study aims to identify disparities and trends in IHD-related mortality rates among obese individuals across various regions and demographics in the US from 2003 to 1999.

We utilized deidentified data from the Centers for Disease Control and Prevention’s Wide-Ranging Online Data for Epidemiologic Research (CDC WONDER) database [4]. A detailed analysis was carried out based on geographic and population factors to assess trends in IHD-related mortality among obese American adults aged 25 and older, spanning from 2003 to 2019. Due to the paucity of data on relevant variables from 1999 to 2002, we analyzed data from 2003 to 2019 to ensure the accuracy of the observed trends. By utilizing the 10th revision of the International Classification of Diseases (ICD-10) codes, we identified all deaths in which IHD (ICD-10 codes: I20–I25) was the underlying cause of death, with obesity (ICD-10 codes: E66.0, E66.1, E66.2, E66.8, and E66.9) as the contributing cause of death. We standardized the crude mortality rates to the 2000 U.S. census population to determine the age-adjusted mortality rates (AAMRs) per 100 000 individuals. To elucidate the long-term mortality trends at the nationwide level, we calculated the annual percent changes (APCs) in AAMRs along with their corresponding 95% confidence intervals (CIs) using the Joinpoint regression model. The Institutional Review Board approval was not sought because we used publicly available deidentified data.

In total, 42 633 770 deaths were recorded between 2003 and 2019. Among these, 6 681 167 were IHD-related only, and 532 747 were obesity-related only. A total of 113 367 deaths were related to IHD in patients with obesity. The AAMR was 175.809 (95% CI: 175.675–175.943) in IHD only, 14.256 (95% CI: 14.217–14.295) in obesity only, and 2.975 (95% CI: 2.957–2.992) in both IHD and obesity (Table 1). The AAMR increased from 2.1 in 2003 to 3.9 in 2019. This increase occurred in three distinct periods: from 2003 to 2009 (APC: 3.39, 95% CI: 0.64–4.38), from 2009 to 2014 (APC: 5.51; 95% CI: 4.56–7.48), and from 2014 to 2019 (APC: 3.00; 95% CI: 1.38–3.79). The elderly group (aged 65 years and older) exhibited the highest AAMR for IHD-related deaths among obese individuals (AAMR: 5.771; 95% CI: 5.716–5.826). This was followed by middle-aged adults (aged 45–64 years) with an AAMR of 4.243 (95% CI: 4.209–4.277) and young adults (aged 25–44 years) with an AAMR of 0.845 (95% CI: 0.829–0.861). Sex stratification revealed a higher rate of IHD-related deaths in patients with obesity in men (AAMR: 3.9; 95% CI: 3.871–3.93) as compared with women (AAMR: 2.102; 95% CI: 2.081–2.122). With stratification by race/ethnicity, the AAMR of IHD-related deaths in patients with obesity was highest in non-Hispanic (NH) Black/African Americans (AAMR: 4.207; 95% CI: 4.142–4.271) followed by NH American Indian/Alaska Native (AAMR: 3.925; 95% CI: 3.678–4.172), NH White (AAMR: 3.063; 95% CI: 3.043–3.084), Hispanic or Latino (AAMR: 1.921; 95% CI: 1.875–1.967), and NH Asian/Pacific Islander population (AAMR: 0.677; 95% CI: 0.638–0.715). Stratification by census region indicated that the Midwest had the highest rate of IHD-related deaths among obese individuals (AAMR: 3.208; 95% CI: 3.169–3.247), followed by the West (AAMR: 3.088; 95% CI: 3.05–3.126), the Northeast (AAMR: 2.862; 95% CI: 2.822–2.901), and the South (AAMR: 2.802; 95% CI: 2.774–2.831). The AAMR categorized by urbanization status revealed a higher rate of IHD-related deaths in patients with obesity in nonmetropolitan areas (AAMR: 3.602; 95% CI: 3.553–3.651) as compared with metropolitan areas (AAMR: 2.874; 95% CI: 2.855–2.893). States such as Vermont, Oklahoma, Wyoming, Iowa, Wisconsin, and the District of Columbia were in the top 90th percentile, whereas Georgia, Maryland, Massachusetts, Connecticut, Virginia, and Alabama were in the lowest 10th percentile (Fig. 1).

**Table 1:** (A) Mortality rates related to ischemic heart disease in patients with obesity and (B) comparison of racial differences among patients who died with ischemic heart disease versus obesity only.

(A) Age-adjusted death rate per 100 000 and frequency for ischemic heart disease in patients with obesity			
	Deaths, *n*	Population, *n*	Overall age-adjusted death rate per 100 000 (95% CI)
Entire cohort	113 367	3 514 312 911	2.975 (2.957–2.992)
Sex			
Men	70 575 (62.25%)	1 693 823 770	3.90 (3.871–3.93)
Women	42 792 (37.75%)	1 820 489 141	2.102 (2.081–2.122)
Race			
Asian	1222	192 565 662	0.677 (0.638–0.715)
Black or African American	17 024	410 000 465	4.207 (4.142–4.271)
American Indian or Alaska Native	1028	26 139 474	3.925 (3.678–4.172)
White	86 261	2 407 188 251	3.063 (3.043–3.084)
Hispanic	7271	478 419 059	1.921 (1.875–1.967)
Census regions of USA			
Northeast	20 713	644 162 067	2.862 (2.822–2.901)
Midwest	26 813	756 312 952	3.208 (3.169–3.247)
South	39 371	1 304 060 203	2.802 (2.774–2.831)
West	26 471	809 777 689	3.088 (3.05–3.126)
Age groups			
Young adults (25–44 years)	10 936	1 424 362 016	0.845 (0.829–0.861)
Middle age (45–64 years)	59 944	1 357 735 687	4.243 (4.209–4.277)
Elderly (65+ years)	42 487	732 215 208	5.771 (5.716–5.826)
(B) Age-adjusted death rate per 100 000 according to race for ischemic heart diseases only and obesity only (95% CI)			
		Ischemic heart disease only	Obesity only
Asian		98.208 (97.684–98.732)	2.674 (2.597–2.75)
Black or African American		205.908 (205.414–206.401)	22.346 (22.197–22.494)
White		179.204 (179.051–179.356)	14.29 (14.244–14.336)
Hispanic		134.629 (134.181–135.076)	9.574 (9.474–9.675)
American Indian or Alaska Native		158.977 (157.139–160.815)	22.887 (22.287–23.487)

CI, confidence interval.

**Fig. 1:**
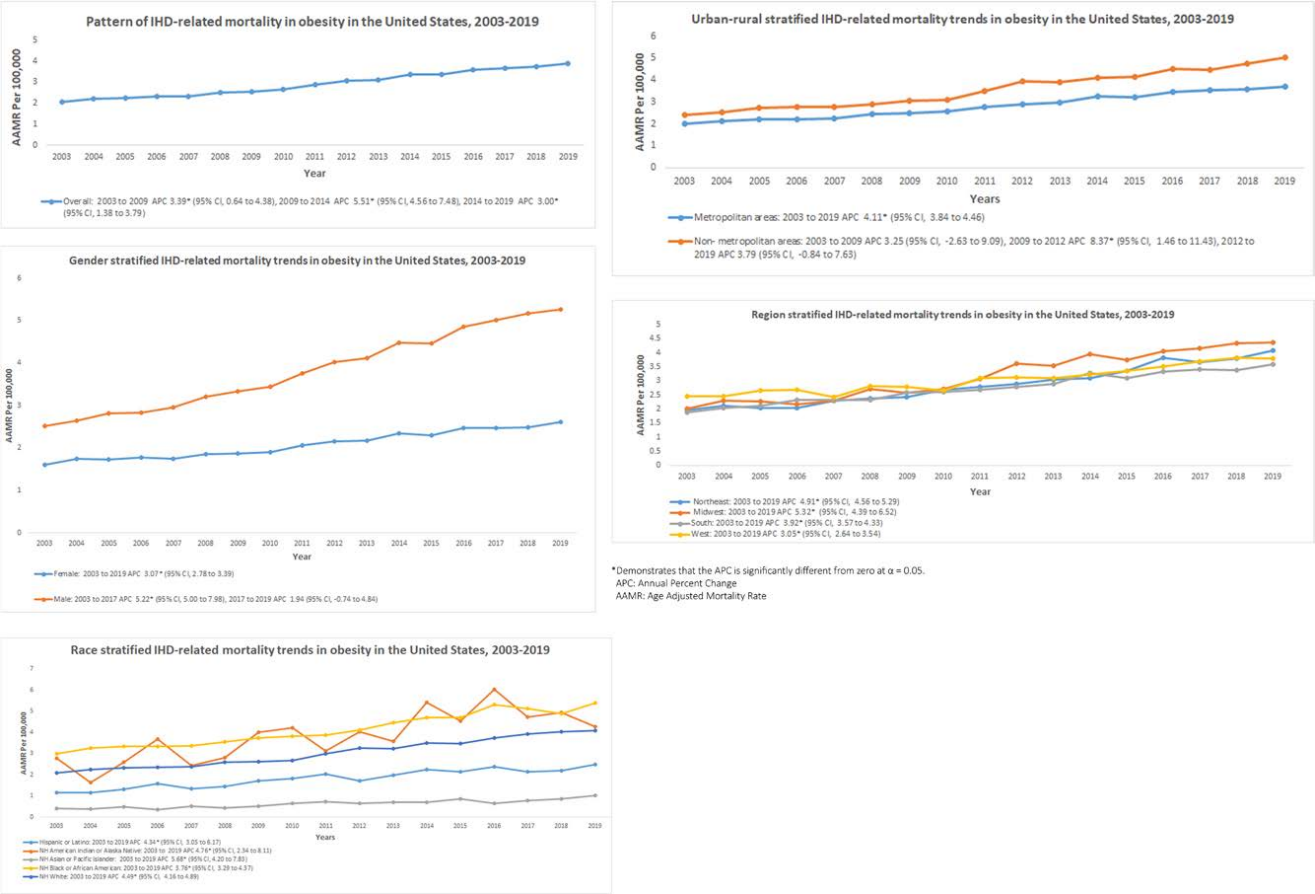
Patterns in age-adjusted death rates per 100 000 for ischemic heart disease among obese individuals, stratified by sex, race, urban–rural, and census region. CI, confidence interval; IHD, ischemic heart disease.

There is a scarcity of data regarding trends in IHD-related mortality among obese individuals, and our study offers valuable insights derived from a large national database. The key findings from our research are as follows:

1. A notable rise in IHD-related mortality among obese adults in the US was observed between 2003 and 2019.
2. Men exhibited higher AAMR than women.
3. Notable differences based on racial status were observed, with NH Black individuals exhibiting higher AAMRs compared with other racial and ethnic groups.
4. Geographical variations were also noteworthy, with the Midwest region showing the highest AAMR and the South region showing the lowest.

Obesity is a leading contributor to IHD, primarily driven by mechanisms such as endothelial dysfunction and dyslipidemia, which accelerate the progression of atherosclerosis [5]. Increased BMI is associated with the reduction of protective high-density lipoprotein cholesterol and elevation of triglycerides and atherogenic low-density lipoprotein cholesterol. Furthermore, increased BMI leads to the transformation of adipose tissues into an endocrine organ, releasing hormones such as leptin, resulting in an increased IHD risk and mortality [5]. In our study, the upward trend in IHD-related mortality among obese individuals may be ascribed to several factors, including the general rise in IHD-related mortality in the US population, the increasing prevalence of obesity in the country, enhanced diagnostic capabilities resulting in more identified cases of IHD, greater awareness among both healthcare providers and patients, and improved documentation of IHD on death certificates for obese patients. Another possible explanation could be the increasing life expectancy in the US, which has resulted in a larger population at risk [6]. This upward trend calls for systematic and strategic management that includes regular cardiovascular surveillance for individuals who are overweight to identify and reduce IHD risk at an early stage. The sex, regional, and racial stratification in our study uncovers significant disparities, emphasizing the necessity for improved policy development, fair distribution of healthcare resources, and further investigation to recognize key areas for tailored interventions.

The outcomes of this study should be viewed with caution due to several indispensable limitations. There is a potential underestimation of mortality rates in the CDC WONDER database, which relies on the accurate reporting and coding of death certificates. The observational design and the lack of more detailed data restricted our capacity to conduct a comprehensive multivariable-adjusted analysis in this study, limiting full understanding of the observed disparities. The database lacks personal records like disease duration, socioeconomic status, specific medical treatments, and comorbidity profiles, which are crucial confounders that influence mortality outcomes. Further research is warranted to address these limitations and provide a more comprehensive understanding of the trends and underlying disparities.

In conclusion, this study indicates that a substantial rise in IHD-related mortality among obese individuals in the US occurred from 2003 to 2019, with elevated mortality rates noted in males and NH Black individuals. These results highlight the necessity for focused interventions to tackle these inequalities and improve health results, ultimately contributing to overall cardiovascular health.

## Acknowledgements

### Conflicts of interest

There are no conflicts of interest.
